# Update on the anatomy of the brachial plexus in dogs: Body weight correlation and contralateral comparison in a cadaveric study

**DOI:** 10.1371/journal.pone.0282179

**Published:** 2023-02-23

**Authors:** Carlotta Lambertini, Margherita De Silva, Annamaria Grandis, Monia Martorelli, Noemi Romagnoli

**Affiliations:** 1 Department of Veterinary Medical Sciences, Alma Mater Studiorum, University of Bologna, Ozzano dell’Emilia, BO, Italy; 2 Private practitioner, Rome, Italy; AIIMS: All India Institute of Medical Sciences, INDIA

## Abstract

A thorough knowledge of the anatomy of the brachial plexus is pivotal for diagnostic, therapeutic and anaesthetic purposes in order to correctly locate the nerve and reduce the incidence of complications when performing surgery or a local anaesthetic block of the brachial plexus. In this study, the anatomy of the brachial plexus in dogs was reviewed; the depth and diameter of each nerve were evaluated, and the contralateral limbs were compared. Eighteen canine cadavers were included and were divided into: small (SB); medium (MB) and large (LB) breed dogs. After dissection, the spinal roots and the suprascapular, subscapular, axillary, radial, ulnar, median, and musculocutaneous nerves were identified. The following evaluations were recorded: the origin of the nerves from the spinal roots, the roots and the nerve diameters, and the distance of the nerves root from the skin at the level of the scapula-humeral joint and from the interscapular region. A total of thirty-six brachial plexuses were evaluated; all originated from the ventral rami of the C6 to T1 spinal nerves. In the LB dogs, the root and the nerve diameters were larger as compared with the other two groups. In this group, also the mean distance of T1 from the skin at the level of the scapula-humeral joint and the average distance of the nerve roots from the skin of the interscapular region were also greater as compared with the other groups. No significant differences were recorded between the contralateral limbs. In the dogs in the present study, the origin of the nerves of the brachial plexus were similar to those previously reported; however, the presence of minor individual variations was confirmed between the right and the left limbs within the same dog between the right and the left limb. This is the first time that the diameters and the depth of the nerves have been described and positively correlated with body weight.

## Introduction

The brachial plexus is a complex anatomical structure consisting of a network formed by the ventral roots of the C4 or C5 spinal nerves up to the T1 or T2 spinal nerves, involved in the formation of the brachial nerves [1]. In past decades, several authors have investigated the anatomy of the brachial plexus in different species. This is the case of the Canidae in which the gross or the ultrasound (US) anatomy of the brachial plexus has been described in the past [2–4]; however, it is still the focus of ongoing research [5]. In fact, it is currently considered to be a structure of “enigmatic complexity” with inter-species and interindividual differences which can probably be ascribed to individual embryonal development [1]. Its particular anatomical variability represents a challenge in the clinical setting when knowledge of its conformation is pivotal for diagnostic purposes, for surgical purposes (i.e after injuries for example) or for anaesthetic purposes (i.e. peripheral nerve blocks) [5]. This is the reason why additional anatomical studies are warranted to better characterise the gross anatomy of the brachial plexus.

In canine patients, locoregional anaesthesia is gaining widespread interest due to its advantages. In fact, properly performed local anaesthetic techniques provide effective intraoperative and postoperative analgesia [6], and a reduction in the inhalant anaesthetic requirement [7]. In addition, when compared with opioid systemic administration, local anaesthesia reduces the surgery associated stress response and the requirement for postoperative analgesic drugs [8].

Regional anaesthesia of the thoracic limb is provided by performing a peripheral nerve block of the brachial plexus [9]. This block can be performed following two main approaches. The technique which first described the brachial plexus location was based on an axillary approach [10], involving the injection of a local anaesthetic solution behind the shoulder, thereby providing desensitisation of the anatomical structure behind the elbow. In 2000, Lemke and Dawson [11] introduced the paravertebral brachial plexus approach which is obtained by blocking the nerves of the brachial plexus at their emergence from the intervertebral foramina as an alternative technique. Compared with the axillary approach, the latter has more distinct anatomical landmarks and provides more proximal pain relief up to the shoulder [7, 11].

Initially, these locoregional blocks had been performed blindly by identifying the anatomical landmarks. In the 1960s, the peripheral nerve stimulator was introduced into the clinical practice as a tool for nerve location and was effective in improving the success rate of the local blocks [12]. Several authors have described the brachial plexus location utilising peripheral nerve stimulation in dogs using either the axillary approach [2, 13, 14] or the paravertebral approach [15]. More recently, ultrasound (US) guided techniques have also been described for blocking the brachial plexus in dogs [15–17]. The introduction of electrolocation-guided and US-guided techniques into clinical practice has increased the accuracy of the brachial plexus block and reduced the incidence of complications, such as intravascular injection and nerve injury. However, the application of these techniques does not reduce the importance of extensive knowledge of the regional anatomy.

In this paper, the gross anatomy of the brachial plexus in dogs was described. With the aim of supplying additional information regarding nerve location while performing the brachial plexus block using a paravertebral approach or an axillary approach, the distance of the nerves forming the brachial plexus from the skin in dogs was investigated. In addition, the diameter of each nerve of the brachial plexus was evaluated in order to facilitate identifying the structure using US examination. Another aim of the study was to evaluate the possible anatomical differences among the contralateral brachial plexuses in dogs.

## Materials and methods

Eighteen canine cadavers obtained from the University Teaching Hospital (University of Bologna, Italy) were used for the anatomical study. The inclusion criteria were cadavers of male and female dogs which had died from or had been euthanised for various medical reasons not involving the forelimbs, the spinal column or the thoracic wall. Cadavers with a body condition score (BCS) ≤ 3/9 or ≥ 6/9 were excluded from the study [18]. The cadavers were frozen and were then thawed completely on the day of the procedure. The dogs were divided into three groups on the basis of their weight. In the group of small breed (SB) dogs, 6 dogs weighing less than 10 kg (1 Pinscher, 1 Dachshund, 1 Yorkshire terrier, 1 Shih Tzu, two mixed breeds) were included. Their mean body weight was 6.8 ± 2.5 kg. In the medium breed (MB) group, 6 dogs weighing between 10 and 20 kg were included (1 Beagle, 1 Breton, 1 Border Collie, three mixed breeds). Their mean body weight was 14.8 ± 3.4 kg. In the large breed (LB) group, 6 dogs weighing more than 20 kg were included (2 German Shepherds, 2 Pointers, 1 Argentine Dogo, 1 mixed breed). Their mean body weight was 27.2 ± 5.5 kg.

No ethical approval was necessary for the use of the canine cadavers. At the end of the study the cadavers were incinerated.

### Dissection technique

**Fig 1:**
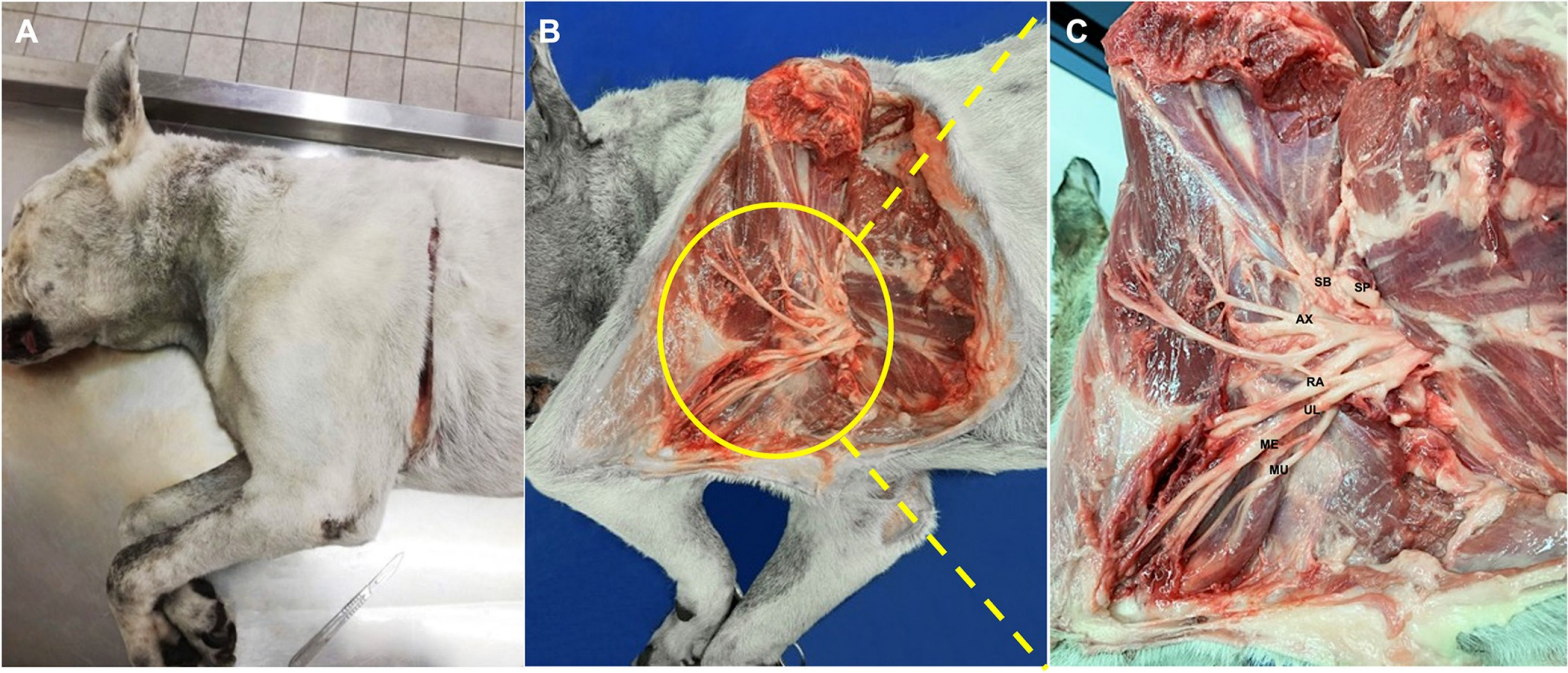
Anatomical dissection. (A) An incision involving the skin, the subcutaneous tissue, the thoracic portion of the trapezius and the *latissimus dorsi* muscles was performed following the caudal limit of the triceps region, proximally following the dorsal margin of the scapula, and distally up to the axillary fold. (B) The forelimb was overturned cranially to expose the brachial plexus at the level of the axillary space. (C) The suprascapular (SP), the subscapular (SB), the axillary (AX), the radial (RA), the ulnar (UL), the median (ME) and the musculocutaneous (MU) nerves were then identified.

The dogs were placed on the anatomical table in left or right lateral recumbency. The anatomical dissection was performed as previously described by Gil and colleagues [19]. An incision involving the skin, the subcutaneous tissue, the thoracic portion of the trapezius and the *latissimus dorsi* muscles was performed following the caudal limit of the triceps region from the caudal scapular angle up to the top of the olecranon process (Fig 1A). The incision was also extended proximally following the dorsal margin of the scapula, and distally up to the axillary fold. In this way, the tissues were dissected and the forelimb was overturned cranially, maintaining the integrity of the nerves (Fig 1B). The brachial plexus at the level of the axillary space was then exposed as was its roots and the nerves directed to the thoracic limb. The suprascapular (SP), subscapular (SB), axillary (AX), radial (RA), ulnar (UL), median (ME) and musculocutaneous (MU) nerves were then identified (Fig 1C). The origins of the different roots of the plexus of each of these nerves and their diameter in mm were evaluated and recorded. The measurements were obtained using a digital caliper (Kennon instruments ®, Sheridan, WY, USA).

**Fig 2:**
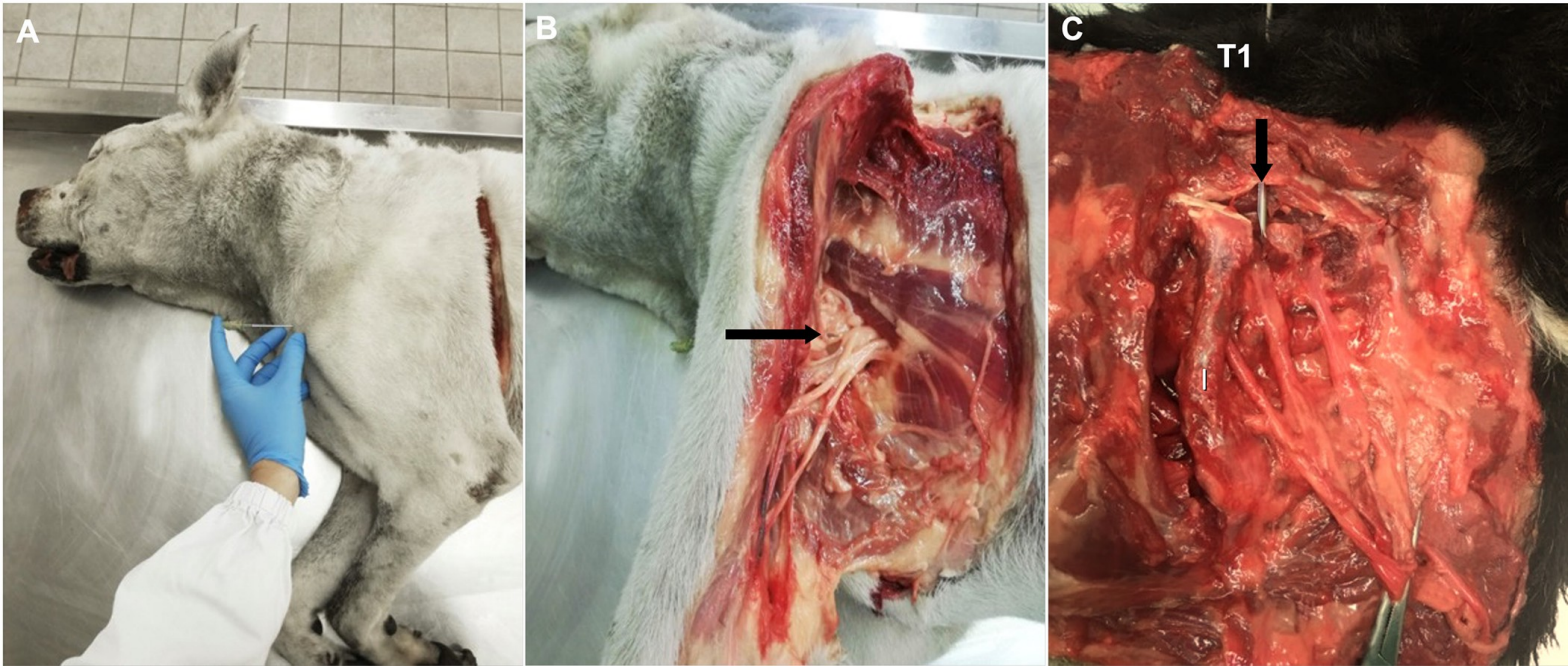
The depth of the nerves forming the brachial plexus was investigated. (A) With the aim of measuring the distance of the T1 from the skin at the level of the shoulder joint, a needle was inserted just above the shoulder tip toward the cranial border of the first rib, parallel to the long axis of the body. (B) The proper positioning of the needle was verified by direct visualisation of the root of the T1, slightly lifting the limb in a craniolateral direction. (C) The limb was removed, and the distance of the nerve roots from the skin of the interscapular region was evaluated. The needle (arrow) was inserted perpendicularly to the vertebral column 2 to 3 cm laterally, and 1 to 2 cm cranially and caudally to the spinous process. T1: indicates the spinous process of the first thoracic vertebra. I: indicates the first rib.

Thereafter, the distance of the root of the T1 from the skin at the level of the shoulder joint was measured and recorded. For this purpose, the limb was repositioned in a physiological position and a needle (16 G, Length 105 mm) was inserted just above the shoulder tip toward the cranial border of the first rib, parallel to the long axis of the body (Fig 2A), following the technique previously described by Campoy and Read [20]. The proper positioning of the needle was verified by direct visualisation of the root of the T1, slightly lifting the limb in a craniolateral direction (Fig 2B). The needle was then drawn back, and the depth of insertion was measured with the aid of a ruler. Subsequently, the limb was removed, and the distance of the nerve roots from the skin of the interscapular region was evaluated (Fig 2C) by inserting a needle following the technique previously described by Lemke and Dawson [11]. In brief, for evaluating the distance of the C8 and the T1 from the skin, a spinal needle was inserted perpendicularly to the vertebral column 2 to 3 cm laterally and 1 to 2 cm cranially and caudally to the spinous process of the first thoracic vertebra in order to reach the roots of the C8 and the T1, respectively. Similarly, for evaluating the distance of the C6 and the C7, the needle was inserted perpendicularly to the vertebral column 2 to 3 cm laterally, and 1 to 2 cm cranially and caudally to the spinous process of the sixth cervical vertebra. The needles were then drawn back, and the depth of insertion was measured with the aid of a ruler.

### Statistical analysis

MedCalc statistical software (MedCalc 6.3 computer software, Belgium) was used for the data analysis. The data were evaluated for normal distribution using a Shapiro-Wilk test. Normally distributed data were reported as mean ± standard deviation (SD). The differences between groups were compared using one-way ANOVA followed by a Tukey–Kramer test. The measurements obtained for the left and right arms were compared using a paired sample T-test. To evaluate the correlation between the weight of the dogs, and the diameters and depth of each nerve, a correlation coefficient (r) was calculated. P*<0*.*05* was considered statistically significant.

## Results

Thirty-six brachial plexuses from eighteen canine cadavers were evaluated. In all the dogs, the brachial plexuses originated from the ventral rami of the C6, C7, C8 and T1 spinal nerves. In addition, the origin of each nerve of the brachial plexus was analysed in detail for both the right and the left arms. The contributions of the spinal roots to the formation of each nerve of the brachial plexus are reported in Table 1.

**Table 1:** Contribution of the spinal roots to the formation of the brachial plexus nerves.

Nerves	Limb Side	Spinal nerves			
		**C6**	**C7**	**C8**	**T1**
**Subscapular**	R	94.4 (17/18)	94.4 (17/18)	5.6 (1/18)	-
	L	88.9 (16/18)	100 (18/18)	-	-
**Suprascapular**	R	100 (18/18)	100 (18/18)	-	-
	L	100 (18/18)	100 (18/18)	-	-
**Axillary**	R	5.6 (1/18)	100 (18/18)	94.4 (17/18)	-
	L	5.6 (1/18)	100 (18/18)	94.4 (17/18)	-
**Radial**	R	-	44.4 (8/18)	100 (18/18)	94.4 (17/18)
	L	-	44.4 (8/18)	100 (18/18)	94.4 (17/18)
**Ulnar**	R	-	-	100 (18/18)	100 (18/18)
	L	-	-	100 (18/18)	100 (18/18)
**Median**	R	-	-	100 (18/18)	100 (18/18)
	L	-	-	100 (18/18)	100 (18/18)
**Musculocutaneous**	R	16.7 (3/18)	94.4 (17/18)	83.3 (15/18)	5.6 (1/18)
	L	5.6 (1/18)	94.4 (17/18)	83.3 (15/18)	11.1 (2/18)

The percentages and numbers (in parentheses) of the right (R) or the left (L) thoracic limbs in which each spinal nerve (from C6 to T1) contributed to the formation of each nerve of the brachial plexus obtained from 18 canine cadavers are reported.

The diameters of the nerve roots and of each nerve are reported in Table 2 as mean and SD. The diameters of the right or the left thoracic limbs did not differ significantly. Overall, the average diameter of the nerve roots and of each nerve of the brachial plexus were greater in the LB dogs as compared with the SB and MB dogs (Table 2).

**Table 2:** Diameters of the nerve roots of the spinal nerves and of the nerves of the brachial plexus of the right (R) or the left (L) thoracic limbs from 18 canine cadavers.

	Limb side	Diameters (mm)			
		SB dogs (n = 6)	MB dogs (n = 6)	LB dogs (n = 6)	ANOVA test (P)
**Nerve Roots**					
C6	R	1.9 ± 0.4^a^	2.2 ± 0.4^ab^	2.7 ± 0.4^b^	0.03
	L	1.9 ± 0.5^a^	2.2 ± 0.5^ab^	2.8 ± 0.4^b^	0.02
		(0.93)	(0.96)	(0.47)	
C7	R	2.9 ± 0.4^a^	3.3 ± 0.4^ab^	3.9 ± 0.6^b^	< 0.01
	L	2.9 ± 0.3^a^	3.2 ± 0.8^a^	4.1 ± 0.5^b^	0.01
		(0.55)	(0.90)	(0.31)	
C8	R	3.3 ± 0.5^a^	3.6 ± 0.4^ab*^	4.2 ± 0.7^b*^	0.03
	L	3.3 ± 0.4^a^	3.4 ± 0.2^a*^	4.5 ± 0.6^b*^	< 0.01
		(0.95)	(0.02)	(0.03)	
T1	R	2.3 ± 0.1^a^	2.8 ± 0.2^ab^	3.3 ± 0.9^b^	0.03
	L	2.4 ± 0.2^a^	2.9 ± 0.3^ab^	3.5 ±0.7^b^	< 0.01
		(0.36)	(0.85)	(0.3)	
**Nerves**					
Subscapular	R	1.2 ± 0.2^a^	1.3 ± 0.2^a^	1.9 ± 0.4^b^	< 0.01
	L	1.2 ± 0.3^a^	1.5 ± 0.4^ab^	1.8 ± 0.6^b^	0.05
		(0.56)	(0.3)	(0.65)	
Suprascapular	R	1.5 ± 0.3^a^	2.2 ± 0.3^ab^	2.5 ± 0.7^b^	0.01
	L	1.7 ± 0.4	2.1 ± 0.2	2.5 ± 0.7	0.06
		(0.33)	(0.50)	(0.48)	
Axillary	R	2.1 ± 0.4^a^	2.6 ± 0.3^ab*^	3.2 ± 0.8^b^	0.01
	L	2.1 ± 0.3^a^	2.4 ± 0.3^ab*^	3.3 ± 1.0^b^	0.01
		(0.81)	(0.04)	(0.6)	
Radial	R	2.4 ± 0.5^a^	3.1 ± 0.4^ab^	3.9 ± 0.8^b^	< 0.01
	L	2.5 ± 0.4^a^	2.9 ± 0.6^ab^	3.9 ± 0.9^b^	< 0.01
		(0.37)	(0.58)	(0.89)	
Ulnar	R	1.7 ± 0.5^a^	1.9 ± 0.5^a^	2.7 ± 0.4^b^	< 0.01
	L	1.7 ± 0.4^a^	1.9 ± 0.4^ab^	2.8 ± 0.8^b^	0.02
		(0.41)	(0.71)	(0.81)	
Median	R	1.3 ± 0.2^a^	1.5 ± 0.1^a^	1.9 ± 0.4^b^	< 0.01
	L	1.3 ± 0.2^a^	1.6 ± 0.2^ab^	2.0 ± 0.4^b^	< 0.01
		(0.76)	(0.14)	(0.75)	
Musculocutaneous	R	1.4 ± 0.3^a^	1.9 ± 0.4^a^	2.5 ± 0.4^b^	< 0.01
	L	1.4 ± 0.3^a^	2.0 ± 0.2^b^	2.5 ± 0.5^b^	< 0.01
		(0.9)	(0.46)	(0.84)	

The data are reported as mean and standard deviation. Values with no symbol did not differ significantly in the ANOVA test or in the T-test. Different superscript letters (a,b) in the same row indicate significant differences between groups in the post hoc analysis. * In the same column significant differences between the R or the L limbs within the same group are reported. The P value of the paired T-tests are reported in the parentheses. P < 0.05 was considered statistically significant.

A positive correlation was found between the roots, the nerve diameters, and the weight of the dogs (S1 and S2 Figs).

The mean distance of the T1 from the skin at the level of the shoulder and the average distance of the nerve roots from the skin of the interscapular region within each group, in the right or in the left arm, did not differ significantly. These measurements were greater in the LB dogs as compared with the other two groups (P < 0.01) (Table 3). A positive correlation was also found between these measurements and the weight of the dogs (S3 Fig).

**Table 3:** Distance of the nerve roots of the spinal nerves from the skin.

		Distance from the skin of the shoulder joint (mm)			
		SB dogs (n = 6)	MB dogs (n = 6)	LB dogs (n = 6)	ANOVA test (P)
T1	R	3.6 ± 0.6 ^a^	5.3 ± 0.3 ^b^	6.4 ± 0.8 ^c^	< 0.01
	L	3.7 ± 0.5 ^a^	5.1 ± 0.5 ^b^	6.3 ± 0.4 ^c^	< 0.01
		(0.77)	(0.37)	(0.75)	
	**Limb side**	**Distance from the skin of the interscapular region (mm)**			
		**SB dogs (n = 6)**	**MB dogs (n = 6)**	**LB dogs (n = 6)**	**ANOVA test (P)**
**Nerve Roots**					
C6	R	4.8 ± 0.8^a^	6.0 ± 0.8^a^	7.5 ± 0.9^b^	< 0.01
	L	5.0 ± 1.1^a^	5.8 ± 0.7^a^	7.6 ± 0.7^b^	< 0.01
		(0.36)	(0.41)	(0.94)	
C7	R	5.3 ± 0.9^a^	6.5 ± 1.1^a^	8.2 ± 0.7^b^	< 0.01
	L	5.3 ± 1.1^a^	6.3 ± 0.8^a^	8.4 ± 0.9^b^	< 0.01
		(0.94)	(0.29)	(0.51)	
C8	R	5.4 ± 0.9^a^	6.7 ± 0.9^b^	8.5 ± 0.6^c^	< 0.01
	L	5.6 ± 0.8^a^	6.5 ± 0.9^a^	8.6 ± 0.8^b^	< 0.01
		(0.41)	(0.22)	(0.71)	
T1	R	5.0 ± 1.0^a^	6.4 ± 0.6^b^	8.1 ± 0.5^c^	< 0.01
	L	5.2 ± 0.9^a^	6.2 ± 0.7^a^	8.1 ± 0.6^b^	< 0.01
		(0.17)	(0.19)	(0.64)	

Data are reported as mean and standard deviation. Values with no symbol did not differ significantly in the ANOVA test or in the T-test. Different superscript letters (a,b,c) in the same row indicate significant differences between groups in the post hoc analysis. * In the same column significant differences between R or L limbs within the same group are reported. The P values of the paired T-tests are reported in the parentheses. P < 0.05 was considered statistically significant.

## Discussion

In the present anatomic study, the contribution of the spinal nerves in the brachial plexus of dogs of different sizes was described. The results of this study confirmed that the ventral branches of the C6 to T1 spinal nerves contributed to the canine brachial plexus while no contribution from the ventral branches of the C5 and T2 spinal nerves was observed. These results were in accordance with those obtained by Skelding and colleagues [21] in their anatomical study on canine cadavers. Previous studies have demonstrated that the ventral branches of the C5 and T2 spinal nerves might also contribute to the formation of the brachial plexus in dogs, even if their contribution was not consistent in all dogs or appeared to be slight, with the most important contribution being provided by C6-T1 [22, 23].

The origin of each nerve was also investigated in the present study. The suprascapular nerve originated from the C6 and C7 in 100% of the limbs considered in accordance with the description of other authors [24–26]. Two different roots [25] can form the subscapular nerve; however, in the present study, this nerve was identified as a single branch originating mainly from the C6 and C7 spinal nerves, as previously reported in dogs [23, 25] with, however, some exceptions. In fact, in the present study, in the right thoracic limb of one dog (Dachshund, SB group), the nerve had its origin from C7 but also from C8; in the right limb of another dog (Beagle, MB group), the nerve had its origin only from C6 while, in the left limbs of two dogs (one Mixed breed and one Argentine Dogo, LB group), the nerve had its origin only from C7. Concerning the origin of the axillary nerve, some authors have previously reported that this nerve had its origin mainly from C7-C8 [25], from C7 only [23] or from C8 only [26]. However, in the present study, it was observed that, in one dog (Beagle, MB group), this same nerve also had its origin from C6. The radial nerve derived mainly from C8 with some branches from C7 and T1 as previously described [23–26]; however, its fibres could also have originated from C6 [25] or from T2 [23, 25]. The origins of the ulnar nerve and median nerve (its median branch in dogs and cats) are in accordance with those reported in previous studies (C8 and T1) [24], with the exception of some contributions from C7 [27] and from T2 [23, 25–27]. The origins of the musculocutaneous nerve varied among species and individuals with a major contribution from C8 and minor contribution from C6, C7, T1 or T2 [25, 27]. Conversely, Allam [23] found that this nerve originated only from C7. In the present study, this nerve originated from C6-T1 with some differences in the contribution of each spinal root between the left and right forelimbs. Moreover, in one dog (Breton, MB group), this nerve originated from T1 only. The results reported herein confirmed the variation existing in the contribution of the nerves in the formation of the brachial plexus. These differences might be individually related or, less likely, breed related. In fact, in the same dog, differences within the contralateral limbs were observed, even if they were minor. These differences could be explained by the theory postulated by other authors who considered that the variation in the formation of the anatomy of the brachial plexus was related to embryonal development. In fact, in this phase, the axon forming the brachial plexus grows out from the spine in an environment in which the arm bud, the growing vascular system and the cartilaginous precursor represent obstacles to its advancement, influencing its final conformation [1].

Knowledge of the origins of each spinal nerve is pivotal in helping the anaesthetist in performing a brachial plexus block using a paravertebral approach, especially when a localised desensitisation of the thoracic limb is required. In fact, a block of the ventral branches of C6-C8 should be sufficient for surgical procedures involving the shoulder and the brachium, and a block of the ventral branches of C7-T1 is indicated for surgical procedures at the elbow, carpus, and digits [7]. A limitation of the present study was that only one dog for each breed was taken into consideration. It cannot be excluded that the inclusion of a larger number of dogs, or of dogs of different breeds and morphotypes, would provide different findings concerning the spinal nerve contributions of the brachial plexus. Additional studies are warranted to evaluate whether there is a breed-related predisposition in the conformation of the brachial plexus. In any case, the operator, when performing the local block, should be aware of the interindividual differences existing in the contribution of each spinal root in the formation of the different nerves of the brachial plexus.

In-depth knowledge of the anatomy is also pivotal for correct nerve location, increasing the success rate and the repeatability of a local anaesthetic block [7] and for reducing the incidence of local block-associated complications [19]. In fact, too deep an advancement of the needle tip over the first rib when performing the brachial plexus block using an axillary approach, might result in pneumothorax or lung laceration [20].

To the Authors’ knowledge, there are no previous studies involving dogs which evaluate the depth of the nerves contributing to the brachial plexus other than information based on the authors’ personal experience [7, 3]. In this study, the depth of each nerve root from the skin was evaluated. The aim of the evaluation of the depth of the T1 from the skin at the level of the shoulder joint was to identify the caudal landmark of the brachial plexus when approached at the axillary level, as previously described by Skelding and colleagues [21]. The procedure carried out for evaluating the distance of the nerve roots from the skin of the interscapular region simulated a paravertebral block of the brachial plexus [7]. As expected, given the conformation and arrangement of the vertebral bodies in the dog, the root farthest away from the skin of the interscapular region was C8 in all the dogs while the closest was C6.

The results demonstrated that, in dogs, for both the paravertebral and the axillary approaches, the depth of the nerves was positively correlated with body weight. These results are helpful for the operator in choosing the ideal needle length and also for understanding how far the tip of the needle should be advanced [3]. The BCS was within the ideal range for all the dogs included in the study; however, a limitation of the study is that the dogs were empirically divided into body size, as is commonly carried out at the Authors’ institution, on the basis of their body weight. This could produce misunderstanding when dealing with dogs differing from the breed standard or with mixed breed dogs. Attention is also recommended in adjusting the depth of the needle insertion if the local anaesthetic block is performed in cachectic or obese animals.

Another aim of the study was to evaluate the diameter of the different nerves; therefore, the average diameters of the small, medium, and large breed dogs have been reported for the first time to be used as reference values. In fact, these results may help in identifying the nerves, especially when performing a US-guided block and even during the learning process of performing the local anaesthetic techniques. A previous study concerning the US anatomy of the canine brachial plexus has already reported the average diameters of the C6-T1 spinal nerves, with the purpose of helping the operators to identify the nerves [4]. In that study, Guilherme and Benigni [4], using Rottweiler and Greyhound cadavers, described the US anatomy of the brachial plexus. They observed that the average diameter of the C6, C7 and C8 spinal nerves at their emergence from the intervertebral foramina was approximately 2.5 mm, and that the average diameter of the musculocutaneous, the median, the ulnar and the radial nerves were approximately 0.5 mm, 1 mm, 1.5 mm, and 0.5–1 mm, respectively. These diameters were smaller as compared with those obtained in the present study, and also as compared with those collected in small breed dogs. In addition, in contrast with these US-guided measurements, the radial nerve has previously been described as the largest of the brachial plexus [23]. Even if in the present study a statistical comparison of the diameters of the different nerves was not carried out, the results highlighted that its diameter was larger as compared with that of the other nerves in all three groups. However, Guilherme and Benigni measured the diameters only using an ultrasound probe and without anatomical dissection [4]. The same authors suggested that the evaluation of nerve diameters was useful when thickening of the nerve was suspected while performing the US-guided block. In those situations, comparison with the US appearance of the contralateral nerve can be performed.

Limb side differences between the contralateral nerves, associated with behaviour or development, have already been reported in animal models using microscopic morphometric observations [28]. Moreover, previous surgical procedures or trauma may affect contralateral fibre characteristics [29]. In male rabbits, significant differences have been observed in the diameters of the fibres of the sciatic nerve between the right and the left limbs [30]. Regarding the aim of the present study, microscopic morphometric evaluation was not carried out, and comparison between the left and the right limbs was limited to the gross anatomy. The results of this study demonstrated that, in dogs, the nerve diameters and the depth of the nerves of the brachial plexus did not differ between the right and the left thoracic limbs. In addition, concerning the contribution of the spinal root to nerve formation, only minor differences were observed between the two thoracic limbs.

The results reported herein were obtained only from cadaveric examination. This was a limitation of the study as the use of frozen and thawed tissues might have slightly influenced the measurements obtained as compared with those which could be measured in vivo. In addition, when evaluating needle advancement, the potential clinical complications associated with the execution of the local block were not assessed.

In conclusion, the present study provided a description of the gross anatomy of the brachial plexus, and of the diameters and the depth of the nerves involved in small, medium, and large breed dogs. The nerve diameters and their depths were positively correlated with the weight of the animals, and the measurements reported did not differ between the right and left arms.

## Supporting information

S1 Fig Correlation between the nerve root diameters and the weight of the dogs. A positive correlation was found between the nerve root diameters and the weight of the dogs. For the C6 root, the correlation coefficient was 0.72 ((P < 0.001; 95% CI 0.39 to 0.89); for the C7 root, the correlation coefficient was 0.68 (P = 0.02 95% CI 0.30 to 0.86); for the C8 root, the correlation coefficient was 0.72 (P< 0.001 95% CI 0.37 to 0.89), and for the T1 root, the correlation coefficient was 0.59 (P = 0.009 95% CI 0.17 to 0.83).
(TIF)

S2 Fig Correlation between the nerve diameters and the weight of the dogs. A positive correlation was found between the nerve diameters and the weight of the dogs. For the subscapular nerve, the correlation coefficient was 0.94 (P< 0.001 95% CI 0.86 to 0.98); for the suprascapular nerve, the correlation coefficient was 0.69 (P = 0.001 95% CI 0.34 to 0.88); for the axillary nerve, the correlation coefficient was 0.69 (P = 0.001 95% CI 0.34 to 0.88); for the median nerve, the correlation coefficient was 0.74 (P = P< 0.001 95% CI 0.42 to 0.89); for the radial nerve, the correlation coefficient was 0.75 (P< 0.001 95% CI 0.43 to 0.9); for the ulnar nerve, the correlation coefficient was 0.68 (P = 0.002 95% CI 0.32 to 0.87), and for the musculocutaneus nerve, the correlation coefficient was 0.83 (P< 0.001 95% CI 0.59 to 0.93).
(TIF)

S3 Fig Correlation between the nerve root depths and the weight of the dogs. The distance of the T1 from the skin calculated at the level of the shoulder joint and the weight of the dogs were positively correlated; the correlation coefficient was 0.91 (P< 0.001 95% CI 0.77 to 0.97). For the C6, the correlation coefficient was 0.88 (P < 0.001 95% CI 0.70 to 0.96); for the C7, the correlation coefficient was 0.86 (P < 0.001 95% CI 0.65 to 0.95); for the C8, the correlation coefficient was 0.89 (P < 0.001 95% CI 0.73 to 0.96), and for T1 the correlation coefficient was 0.88 (P < 0.001 95% CI 0.71 to 0.96).
(TIF)

S1 Table Original data. The tables display the original data concerning the diameters of the nerve roots of the spinal nerves and of the nerves of the brachial plexus and the distance of the nerve roots of the spinal nerves from the skin of the right (R) or the left (L) thoracic limbs from 18 canine cadavers. In addition, the contribution of the spinal roots to the formation of the brachial plexus nerves is reported.
(XLSX)

## References

[1] Leijnse JN, de Bakker BS, D’Herde K. The brachial plexus–explaining its morphology and variability by a generic developmental model. J Anat. 2020;236: 862–882. doi: 10.1111/joa.13123 31814126 PMC7163732

[2] Mahler SP, Adogwa AO. Anatomical and experimental studies of brachial plexus, sciatic, and femoral nerve-location using peripheral nerve stimulation in the dog. Vet Anaesth Analg. 2008;35: 80–89. doi: 10.1111/j.1467-2995.2007.00356.x 17696969

[3] Hofmeister EH, Kent M, Read MR. Paravertebral block for forelimb anesthesia in the dog–an anatomic study. Vet Anaesth Analg. 2007;34: 139–142. doi: 10.1111/j.1467-2995.2006.00313.x 17316395

[4] Guilherme S, Benigni L. Ultrasonographic anatomy of the brachial plexus and major nerves of the canine thoracic limb. Vet Radiol Ultrasound. 2008;49: 577–583. doi: 10.1111/j.1740-8261.2008.00424.x 19051650

[5] Grzeczka A, Zdun M. The Structure of the Brachial Plexus in Selected Representatives of the Caniformia Suborder. Animals (Basel). 2022;12(5): 566. doi: 10.3390/ani12050566 35268135 PMC8908818

[6] De Marzo C, Crovace A, De Monte V, Grimaldi D, Iarussi F, Staffieri F. Comparison of intra-operative analgesia provided by intravenous regional anesthesia or brachial plexus block for pancarpal arthrodesis in dogs. Res Vet Sci. 2012;93: 1493–1497. doi: 10.1016/j.rvsc.2012.03.001 22464864

[7] Lemke KA, Creighton CM. Paravertebral blockade of the brachial plexus in dogs. Vet Clin North Am Small Anim Pract. 2008;38: 1231–vi. doi: 10.1016/j.cvsm.2008.06.003 18954682

[8] Romano M, Portela DA, Breghi G, Otero PE. Stress-related biomarkers in dogs administered regional anaesthesia or fentanyl for analgesia during stifle surgery. Vet Anaesth Analg. 2016;43: 44–54. doi: 10.1111/vaa.12275 25996102

[9] Wenger S, Moens Y, Jäggin N, Schatzmann U. Evaluation of the analgesic effect of lidocaine and bupivacaine used to provide a brachial plexus block for forelimb surgery in 10 dogs. Vet Rec. 2005;156: 639–42. doi: 10.1136/vr.156.20.639 15894729

[10] Nutt P. Brachial plexus analgesia in the dog. Vet Rec. 1962;74: 874–876.

[11] Lemke KA, Dawson SD. Local and regional anaesthesia. Vet Clin North Am Small Anim Pract. 2000;30: 839–857. 10932828 10.1016/s0195-5616(08)70010-x

[12] Greenblatt GM, Denson JS. Needle nerve stimulatorlocator: nerve blocks with a new instrument for locating nerves. Anesth Analg. 1962;41: 599–602. 13901498

[13] Futema F, Fantoni DT, Auler JO Jr, Cortopassi SR, Acaui A, Stopiglia AJ. A new brachial plexus block technique in dogs. Vet Anaesth Analg. 2002;29: 133–139. doi: 10.1046/j.1467-2995.2002.00082.x 28404237

[14] Akasaka M, Shimizu M. Comparison of ultrasound- and electrostimulation-guided nerve blocks of brachial plexus in dogs. Vet Anaesth Analg. 2017;44: 625–635. doi: 10.1016/j.vaa.2016.08.001 28624495

[15] Rioja E, Sinclair M, Chalmers H, Foster RA, Monteith G. Comparison of three techniques for paravertebral brachial plexus blockade in dogs. Vet Anaesth Analg. 2012;39: 190–200. doi: 10.1111/j.1467-2995.2011.00677.x 22117891

[16] Bagshaw HS, Larenza MP, Seiler GS. A technique for ultrasound-guided paravertebral brachial plexus injections in dogs. Vet Radiol Ultrasound. 2009;50: 649–654. doi: 10.1111/j.1740-8261.2009.01599.x 19999352

[17] Monticelli P, Fitzgerald E, Viscasillas J. A sonographic investigation for the development of ultrasound-guided paravertebral brachial plexus block in dogs: cadaveric study. Vet Anaesth Analg. 2018;45: 195–202. doi: 10.1016/j.vaa.2017.08.005 29398529

[18] WSAVA Nutritional Assessment Guidelines Task Force Members, Freeman L, Becvarova I, et al. WSAVA Nutritional Assessment Guidelines. J Small Anim Pract. 2011;52: 385–396. 21649660 10.1111/j.1748-5827.2011.01079.x

[19] Gil J, Gimeno M, Laborda J, Nuviala J. Anatomía del perro–protocolos de disección. Barcelona: Ed. Masson; 1997.

[20] Campoy Campoy L and Read MR. The thoracic limb. In: Campoy L, Read MR, editors. Small animal regional anesthesia and analgesia. Iowa: Blackwell Publishing; 2013. Pp. 141–198.

[21] Skelding A, Valverde A, Sinclair M, Thomason J, Moens N. Anatomical characterization of the brachial plexus in dog cadavers and comparison of three blind techniques for blockade. Vet Anaesth Analg. 2018;45: 203–211. doi: 10.1016/j.vaa.2017.11.002 29366667

[22] Miller RA. Comparative studies upon the morphology and distribution of the brachial plexus. J Nerv Ment Dis. 1934;80: 84.

[23] Mw Allam, Dg Lee, Fe Nulsen, Ea Fortune. The anatomy of the brachial plexus of the dog. Anat Rec. 1952;114: 173–179. doi: 10.1002/ar.1091140205 12986272

[24] König HE, Liebich HG. Veterinary anatomy of domestic mammals: textbook and colour atlas 7th ed. New York: Schattauer; 2020.

[25] Barone R, Simoens P. Anatomie compare des mammifères domestiques. Neurologie II. Systeme nerveux périférique, glandes endocrines, esthésiologie. Paris: Ed. Vigot; 2010.

[26] Dyce Singh B., Sack, and Wensing’s Textbook of veterinary anatomy. 5th ed. St. Louis: Elsevier; 2018.

[27] Sharp JW, Bailey CS, Johnson RD, Kitchell RL. Spinal nerve root origin of the median, ulnar and musculocutaneous nerves and their muscle nerve branches to the canine forelimb. Anat Histol Embryol. 1990;19: 359–368. doi: 10.1111/j.1439-0264.1990.tb00911.x 2077954

[28] Christensen MB, Tresco PA. Differences Exist in the Left and Right Sciatic Nerves of Naïve Rats and Cats. Anat Rec. 2015;298: 1492–1501. 10.1002/ar.23161 25857635

[29] Tamaki K. Further studies on the effect of section of one peroneal nerve of the albino rat on the intact nerve of the opposite side. J. Comp. Neurol. 1936;64: 437–448.

[30] Muglia U, Vita G, Laura R, Mammola CL, Germana G. Morphometric comparison between contralateral sciatic nerves in the male and female rabbit. Anat Histol Embryol. 1997;26: 147. doi: 10.1111/j.1439-0264.1997.tb00115.x 9210786

