# Supplementary figures and images for: Update on the anatomy of the brachial plexus in dogs: Body weight correlation and contralateral comparison in a cadaveric study
Source: PLoS One. 2023 Feb 23;18(2):e0282179. doi: 10.1371/journal.pone.0282179 (PMC9949655; doi:10.1371/journal.pone.0282179)

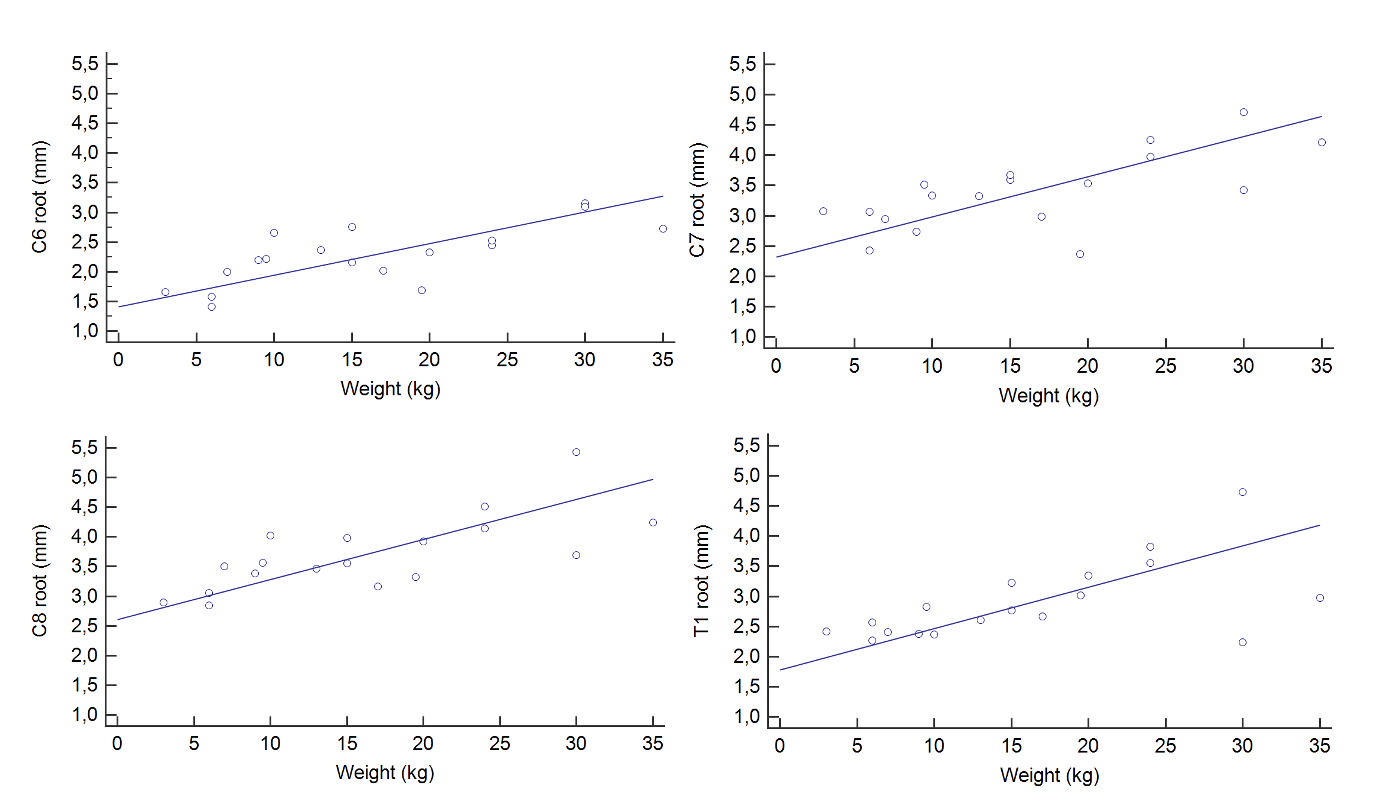

Supplement: S1 Fig — A positive correlation was found between the nerve root diameters and the weight of the dogs. For the C6 root, the correlation coefficient was 0.72 ((P < 0.001; 95% CI 0.39 to 0.89); for the C7 root, the correlation coefficient was 0.68 (P = 0.02 95% CI 0.30 to 0.86); for the C8 root, the correlation coefficient was 0.72 (P< 0.001 95% CI 0.37 to 0.89), and for the T1 root, the correlation coefficient was 0.59 (P = 0.009 95% CI 0.17 to 0.83). (TIF) [file pone.0282179.s001.tif]

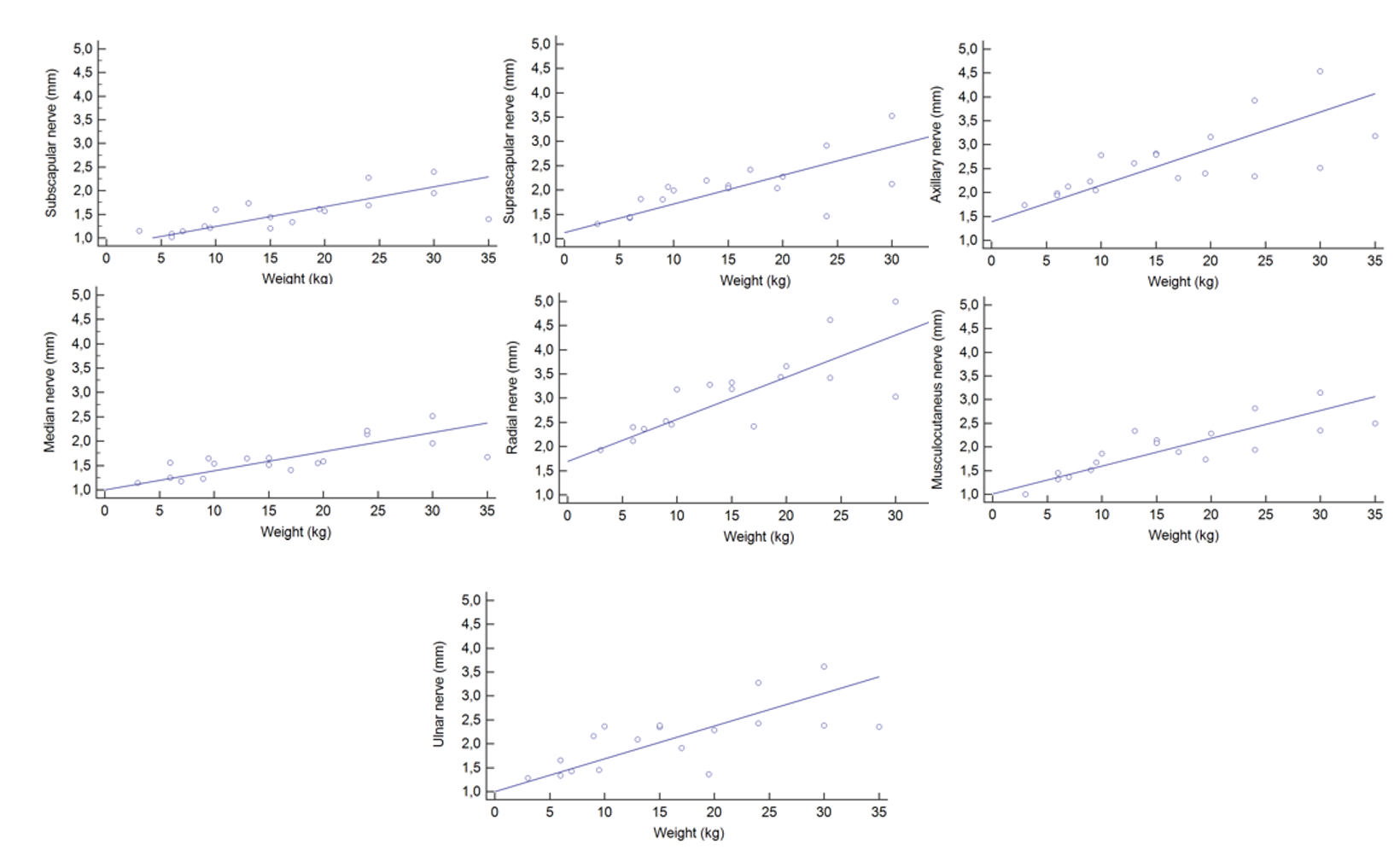

Supplement: S2 Fig — A positive correlation was found between the nerve diameters and the weight of the dogs. For the subscapular nerve, the correlation coefficient was 0.94 (P< 0.001 95% CI 0.86 to 0.98); for the suprascapular nerve, the correlation coefficient was 0.69 (P = 0.001 95% CI 0.34 to 0.88); for the axillary nerve, the correlation coefficient was 0.69 (P = 0.001 95% CI 0.34 to 0.88); for the median nerve, the correlation coefficient was 0.74 (P = P< 0.001 95% CI 0.42 to 0.89); for the radial nerve, the correlation coefficient was 0.75 (P< 0.001 95% CI 0.43 to 0.9); for the ulnar nerve, the correlation coefficient was 0.68 (P = 0.002 95% CI 0.32 to 0.87), and for the musculocutaneus nerve, the correlation coefficient was 0.83 (P< 0.001 95% CI 0.59 to 0.93). (TIF) [file pone.0282179.s002.tif]

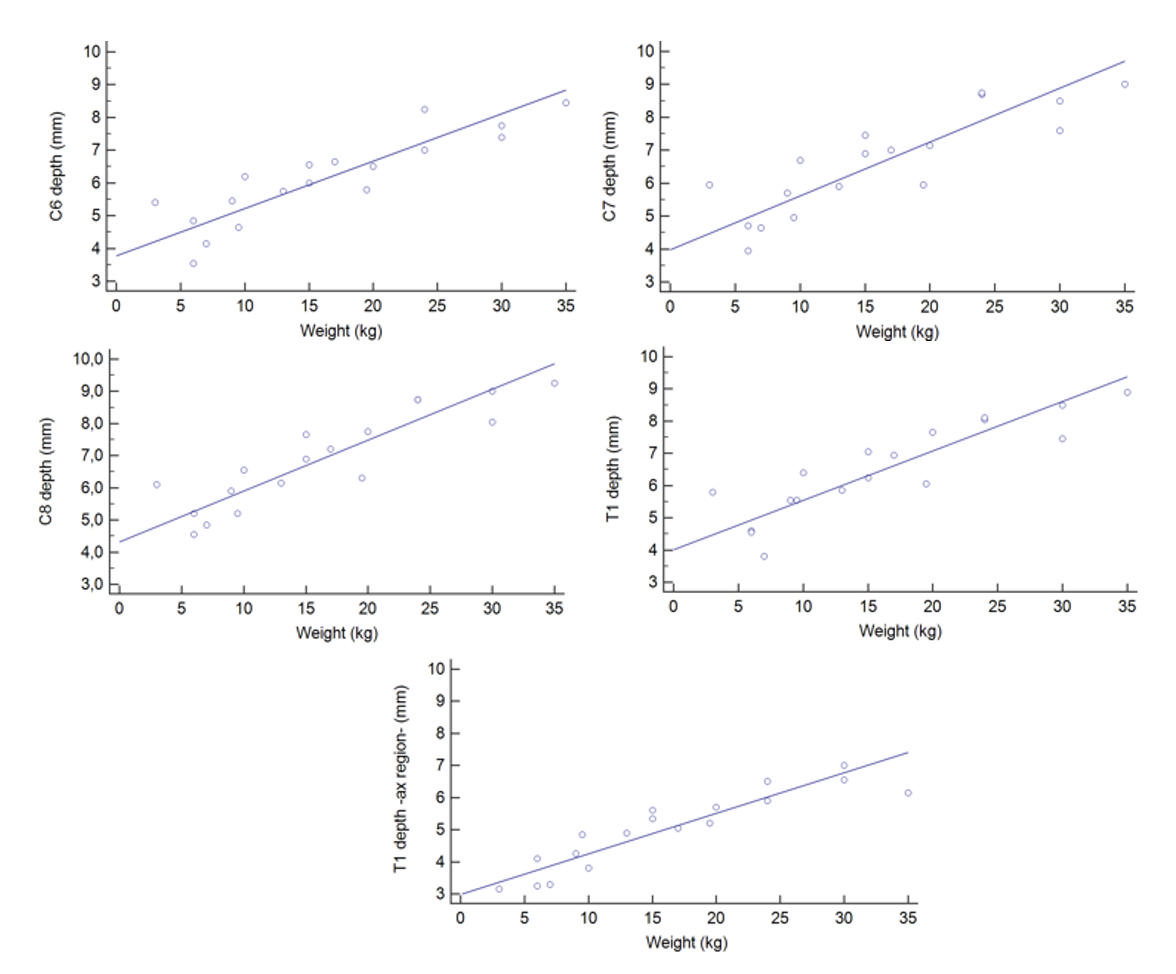

Supplement: S3 Fig — The distance of the T1 from the skin calculated at the level of the shoulder joint and the weight of the dogs were positively correlated; the correlation coefficient was 0.91 (P< 0.001 95% CI 0.77 to 0.97). For the C6, the correlation coefficient was 0.88 (P < 0.001 95% CI 0.70 to 0.96); for the C7, the correlation coefficient was 0.86 (P < 0.001 95% CI 0.65 to 0.95); for the C8, the correlation coefficient was 0.89 (P < 0.001 95% CI 0.73 to 0.96), and for T1 the correlation coefficient was 0.88 (P < 0.001 95% CI 0.71 to 0.96). (TIF) [file pone.0282179.s003.tif]
